# Spatiotemporal evolution of coronavirus disease 2019 mortality in Brazil in 2020

**DOI:** 10.1590/0037-8682-0282-2020

**Published:** 2020-06-01

**Authors:** Carlos Dornels Freire de Souza, Gibson Barros de Almeida Santana, Thiago Cavalcanti Leal, João Paulo Silva de Paiva, Leonardo Feitosa da Silva, Lucas Gomes Santos, Michael Ferreira Machado, Divanise Suruagy Correia, Victor Santana Santos, Rodrigo Feliciano do Carmo

**Affiliations:** 1 Universidade Federal de Alagoas, Departamento de Medicina, Arapiraca, AL, Brasil.; 2 Universidade Federal de Alagoas, Departamento de Medicina, Maceió, AL, Brasil.; 3 Universidade Federal de Alagoas, Centro de Epidemiologia e Saúde Pública, Arapiraca, AL, Brasil.; 4 Universidade Federal do Vale do São Francisco, Programa de Pós-Graduação Ciências da Saúde e Biológicas, Petrolina, PE, Brasil.; 5 Universidade Federal do Vale do São Francisco, Pós-Graduação em Biociências, Petrolina, PE, Brasil.

**Dear Editor,**

Coronavirus disease 2019 (COVID-19) is a highly infectious disease that emerged in December 2019 in China^1^ and has spread rapidly worldwide^2^. Globally, COVID-19 infected more than 3.1 million people and caused more than 220,000 deaths in the first quarter of 2020^2^ ^,^ ^3^.

The first case in Brazil was recorded on February 26, 2020, and the first death on March 17, 2020^4^. Soon after the disease was detected in Brazil, the number of cases and deaths increased daily, and 2 months later (April 29, 2020), Brazil had 71,866 cases and 5,017 deaths, even more than China, the origin of the pandemic^5^.

Monitoring mortality from COVID-19 plays a fundamental role in assessing severity and may serve as a tool for decision making. This study analyzed the spatiotemporal distribution of COVID-19 mortality in Brazilian states, between March 17, 2020, and April 24, 2020 (epidemiological weeks [EWs] from 12 to 17).

This was an ecological study involving all deaths from COVID-19. Geographic units of the analysis were the state and EW. Data were obtained from the Ministry of Health of Brazil (https://covid.saude.gov.br) on April 25, 2020, and population data were obtained from the Brazilian Institute of Geography and Statistics (https://www.ibge.gov.br/). Accumulated mortality rates for each state and EW were calculated using the following equation:

$$\text{Mortality rate =}\frac{Number\;of\;deaths\;per\;COVID-19\;case\;}{Resident\;population\;in\;the\;year}\;\times \;1\;million\;$$

In addition, we performed an exploratory spatial analysis, with stratification at equal intervals. Choropleth maps were prepared to show the results. As we used secondary data from the public domain, the Research Ethics Committee's approval was waived.

The first death from COVID-19 was registered in São Paulo on March 17, 2020. Two days later (March 19, 2020), two deaths were confirmed in Rio de Janeiro. In EW 12, both São Paulo and Rio de Janeiro accounted for 18 deaths together. In EW13, 10 states had already reported deaths, and São Paulo (1.82/1 million) and Rio de Janeiro (0.77/1 million) had the highest rates. Together, both states accounted for 97 out of 114 deaths recorded in Brazil. In EW 14, São Paulo (5.62/million), Rio de Janeiro (3.42/1 million), Amazonas (2.83/1 million), and Ceará (2.40/1 million) had the highest mortality rates. In EW 15, Amazonas had the highest mortality rate among all states (12.50/1 million), followed by São Paulo (12.26/1 million). In the same EW, Pernambuco experienced a significant increase in mortality (from 1.45/1 million to 7.46/1 million). In EW 16, the mortality rate was tripled in Amazonas, reaching 37.9/1 million. The mortality in Amazonas was 1.7 times higher than that in São Paulo (21.51/1 million). In EW 17, the highest COVID-19 mortality was in Amazonas at 60.14 deaths/1 million (n = 255 deaths), followed by Pernambuco (36.47/1 million), Rio de Janeiro (33.64/1 million), São Paulo (32.82/1 million), and Ceará (30.94/1 million).

This study showed the dynamics of the evolution of death rates by Brazilian states in the first six EWs of COVID-19 mortality in Brazil **(** Figure 1 **)**. The first cases and deaths were reported in São Paulo and Rio de Janeiro. As soon as cases of death in São Paulo and Rio de Janeiro emerged, surveillance services in other states began to report both cases and deaths. A few weeks later, North and Northeast states were the ones with the highest mortality rates.

**FIGURE 1:**
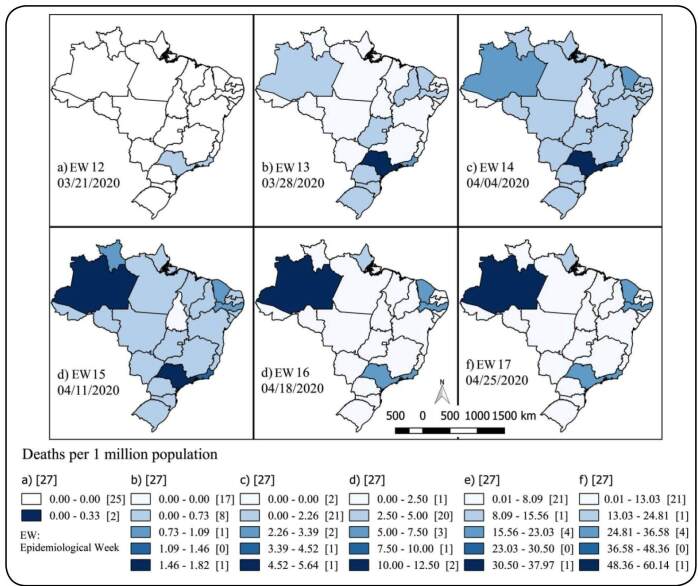
Spatiotemporal evolution of the coronavirus disease 2019 mortality rate (per 1 million inhabitants) in Brazil between the epidemiological weeks 12 and 17 of 2020.

Obviously, since the community transmission of COVID-19 has commenced, tracing a faithful route for distribution of cases has become difficult. However, some explanations can be given to characterize the spread of COVID-19 in Brazil. São Paulo and Rio de Janeiro are considered Brazil's “entrance gates” because of the concentration of international passengers and cargo flights, and most flights from Europe and the United States arrive in São Paulo or Rio de Janeiro^6^. In addition, São Paulo and Rio de Janeiro have the highest concentration of domestic flights in the country, operated either as arrival/departure or as connecting points, thus likely to be responsible for the spread of the disease to other states^6^.

Although both air transport and traffic network are important to understand the dynamics of COVID-19 spread across the country, it alone cannot explain the differences in mortality rates among states. Preliminary reports have shown that COVID-19 mortality has been associated with old age and comorbidities, such as cardiovascular disease and diabetes^7^ ^-^ ^9^. However, mortality rates vary widely even in these population groups. Our study found that on April 24, 2020, the mortality rate in Amazonas was almost twice as high as mortality that in São Paulo or Rio de Janeiro. This suggests that social and demographic factors and supportive differences in health services may play an important role as factors associated with COVID-19 mortality.

As COVID-19 progresses rapidly to severe pneumonia and acute respiratory distress syndrome^8^, critically ill patients need mechanical ventilatory support at intensive care units (IUCs). Thus, the varying availability of such resources can be an additional risk factor for death from COVID-19. Amazonas has 6.5 adult ICU beds for every 100,000 people, which is 3.3 times less than those in Rio de Janeiro (21.9/100,000 population) and 2.7 times less than those in São Paulo (18.0/100,000 population). When only public health beds are analyzed, the availability in Amazonas is lower (5.0/100,000 population)^10^.

An estimated 22.6% of people who are exclusively in the public health system have no access to ICU beds at their resident health regions, as these ICU beds are concentrated in the metropolitan regions. Additionally, 9.8% of health regions have 10 beds per 100,000 population^10^. Regarding human resources, the region contains only 4% of the total number of physicians working in the country, which represents approximately one physician for every 1,000 inhabitants. This means seven times less than the availability of physicians in the capitals from states in the South region^11^. The situation is even more serious in municipalities far from the capital. For example, in Amazonas, the capital Manaus has a rate of two physicians per 1,000 inhabitants, while in municipalities in the interior of Amazonas, the rate is 10 times lower (0.2/1,000 inhabitants)^11^ ^,^ ^12^. Thus, it is likely that the scarcity of human and material resources for health care associated with the rapid spread of COVID-19 may contribute to increased mortality.

In the Northeast region, Pernambuco and Ceará presented with high COVID-19 mortality. Similar to the North region, the Northeast region faced social vulnerability, since 30.5% of people who are exclusively in the public health system have no access to ICU beds in their residence health regions^10^. In addition, all states in the Northeast region have less than two physicians per 1,000 inhabitants^12^.

This study has some limitations. As we analyzed secondary data from surveillance systems, it is likely that COVID-19 deaths were underreported. Brazil is one of the countries performing the least number of tests for COVID-19 worldwide. However, underreporting is likely to occur randomly and has not influenced our findings.

In summary, Brazilian states with the greatest lack of health resources have shown the highest COVID-19 mortality rate. Inequalities in availability and access to the health care system represent additional challenges, given the increase in COVID-19 mortality in the country. This study reinforces the need for an urgent expansion of the operational capacity of the Unified Health System.

## References

[1] Zhu N, Zhang D, Wang W, Li X, Yang B, Song J (2020). A Novel Coronavirus from Patients with Pneumonia in China, 2019. N Engl J Med.

[2] World Health Organization (2020). Coronavirus disease 2019 (COVID-19): Situation Report - 51.

[3] Johns Hopkins University (2020). COVID-19 Dashboard by the Center for Systems Science and Engineering (CSSE).

[4] Brasil (2020). Boletim Epidemiológico: Situação epidemiológica da COVID-19 - Doença pelo coronavírus n 09.

[5] Brasil Coronavirus Brasil.

[6] Brasil. Ministério dos Transportes, Portos e Aviação Civil (2018). Plano Aeroviário Nacional 2018.

[7] Zhang J, Yu M, Tong S, Liu LY, Tang LV (2020). Predictive factors for disease progression in hospitalized patients with coronavirus disease 2019 in Wuhan, China. J Clin Virol..

[8] Martins-Filho PR,  Tavares CSS,  Santos VS (2020). Factors Associated With Mortality in Patients With COVID-19. A Quantitative Evidence Synthesis of Clinical and Laboratory Data. Eur J Intern Med.

[9] Sarasin F, Dami F, Carron PN (2020). Emergency Medical Services: COVID-19 crisis. Rev Med Suisse..

[10] Rache B, Rocha R, Nunes L, Spinola P, Malik AM, Massuda A (2020). Necessidades de Infraestrutura do SUS em Preparo à COVID-19: Leitos de UTI, Respiradores e Ocupação Hospitalar.

[11] Garnelo L, Lima JG, Rocha ESC, Herkrath FJ (2018). Acesso e cobertura da Atenção Primária à Saúde para populações rurais e urbanas na região norte do Brasil. Saúde Debate.

[1] Scheffer M, Cassenote A, Guilloux AGA, Biancarelli A, Mioto BA, Mainardi GM (2018). Demografia Médica no Brasil 2018.

